# A Review on the Study of Cyanotoxins in Paleolimnological Research: Current Knowledge and Future Needs

**DOI:** 10.3390/toxins12010006

**Published:** 2019-12-20

**Authors:** Eliana Henao, Piotr Rzymski, Matthew N. Waters

**Affiliations:** 1 Department of Biology, Universidad del Valle, 100-00 Cali, Colombia; 2 Department of Environmental Medicine, Poznan University of Medical Sciences, 60-806 Poznan, Poland; 3 Department of Crop, Soil and Environmental Sciences, Auburn University, Funchess Hall, AL 36849, USA

**Keywords:** cyanobacteria, paleolimnology, cyanotoxins, microcystins, cylindrospermopsin

## Abstract

Cyanobacterial metabolites are increasingly studied, in regards to their biosynthesis, ecological role, toxicity, and potential biomedical applications. However, the history of cyanotoxins prior to the last few decades is virtually unknown. Only a few paleolimnological studies have been undertaken to date, and these have focused exclusively on microcystins and cylindrospermopsins, both successfully identified in lake sediments up to 200 and 4700 years old, respectively. In this paper, we review direct extraction, quantification, and application of cyanotoxins in sediment cores, and put forward future research prospects in this field. Cyanobacterial toxin research is also compared to other paleo-cyanobacteria tools, such as sedimentary pigments, akinetes, and ancient DNA isolation, to identify the role of each tool in reproducing the history of cyanobacteria. Such investigations may also be beneficial for further elucidation of the biological role of cyanotoxins, particularly if coupled with analyses of other abiotic and biotic sedimentary features. In addition, we identify current limitations as well as future directions for applications in the field of paleolimnological studies on cyanotoxins.

## 1. Introduction

Cyanobacterial metabolites are receiving increased attention in regards to their chemistry, biosynthesis, ecological role, toxicity, and potential biomedical use [1,2,3,4,5]. The major compounds of concern include hepatotoxic microcystins and nodularins; cytotoxic cylindrospermopsin; and neurotoxic anatoxin-a, saxitoxins, and beta-methylamino-L-alanine. These cyanobacterial toxins can impact aquatic organisms, as well as pose a threat to humans and terrestrial animals [6,7]. While the focused concern with these compounds started quite recently (<20 years) from a scientific standpoint, it is plausible that their biosynthesis evolved a relatively long time ago. Initially, it was suggested that toxin synthesis developed primarily as a chemical defense mechanism against planktivores [8,9]. However, research suggests that they likely predated metazoan lineage, which diverged approximately 1.5 billion years ago [10]. For example, syntenic gene analysis and phylogenetic investigation of primary sequences indicate that the gene cluster responsible for saxitoxin production in early Nostocales has been present for at least 2.1 billion years [11]. Phylogenetic evidence for the early evolution of microcystin synthesis has also been identified [12], and indirect cues for similar phenomena in the case of cylindrospermopsin production also exist [3]. These timescales imply that cyanobacterial toxins play ecological role(s) other than grazer defense, and that their toxicity towards zooplankton may only be an indirect effect of their production and release into the water column. Cyanotoxin biological function still remains a subject of debate and various hypotheses, derived mostly from experimental observations, on their potential intra- and extracellular roles have been put forward [1,12,13,14].

Like the ecological role of cyanobacterial metabolites, the environmental triggers causing toxin production lack definite identification in experimental and monitoring investigations. Nutrients, frequently associated with cultural eutrophication (nitrogen and phosphorus) in freshwaters, have been identified as key drivers of toxin production [15], but cyanobacteria in selected hypereutrophic systems do not produce cyanotoxins [1]. Nutrients that generally have less influence on trophic state in freshwater ecosystems, such as S and Fe, have also been associated with toxin occurrence [16], as have changes in N/P ratios [15]. Biological drivers, such as zooplankton grazing pressure and allelopathy [1], have been linked to cyanobacteria toxin production; along with abiotic factors, such as temperature and light intensity [17,18]. While recent genetic, experimental, and monitoring efforts have provided extensive knowledge of cyanobacterial metabolites, placing these data into a historic context and determining whether toxin production is a modern phenomenon has received less attention. One way to provide historic reconstructions of toxin production, in addition to previous ecological triggers, is to utilize the sediment record of aquatic systems and apply paleolimnological tools.

Paleolimnology uses sediment cores collected from aquatic environments to reconstruct a history of natural changes preserved in physical, chemical, and biological features [19]. Cyanobacterial occurrence has been inferred by utilizing a number of approaches mostly based on pigment quantification, counting of akinetes (which only applies to filamentous species classified in the orders *Nostocales* and *Stigonematales*), and more recently, sequencing of ancient DNA [20,21,22,23,24]. Although these methods are valuable in tracking trends of past cyanobacterial abundance, paleolimnological investigations on the history and dynamics of cyanotoxin occurrence have yet to be established on a broad scale. This can be achieved by obtaining direct cyanotoxin measurements of sediment extractions, or analysis of DNA sequences isolated from sediments. One should note, however, that sediment aging is associated with gradual DNA degradation, demonstrated by strand breakage, miscoding lesions, abasic sites, and crosslinks [24,25,26,27]. Consequently, a number of amplifiable templates are decreased while the risk of sequencing artifacts is high. Some research has successfully analyzed 500-bp-long-fragments in sediments dated at 10,000 years before present (YBP) [28], although a number of studies have shown that usually only shorter fragments, isolated from much younger sediments, can be amplified [29,30]. Nevertheless, sedimentary DNA can still be a valuable tool in reconstructing the composition of various groups of aquatic organisms, including cyanobacteria, and can provide a historical overview on the distribution of cyanotoxin producers [24].

Interestingly, only a few studies have attempted to directly quantify cyanobacterial metabolites in lake sediment cores (Table 1). While some metabolites may be prone to degradation prior to accumulation in lake depositional areas, some toxic cyanobacterial compounds, such as cylindrospermopsin and microcystins, have proven to be relatively stable under varying environmental conditions [31,32]; these metabolites are ideal candidates for paleolimnological studies. Moreover, the quantification of cyanotoxins in sediments can potentially provide an insight into the ecological role of these compounds if coupled with analyses of other abiotic and biotic features, such as nutrients or subfossil-zooplankton records [33]. Finally, coupling amplification of ancient DNA with direct analyses of the sedimentary levels of cyanotoxins could also provide an overview on the occurrence of these metabolites in the past, and potential species responsible for their production.

This paper reviews studies which have reconstructed cyanotoxin production from sediment cores, identifies current limitations in analytical methods, and outlines future research prospects. We hope that this will spark increased interest in research focused on tracking the history and dynamics of cyanobacteria, and in new data which may help elucidate the ecological role of cyanotoxins and the environmental triggers of their production.

## 2. Determination of Cyanotoxins in Sediment Cores

Paleolimnological investigations of cyanotoxins have focused on microcystins (MCs) and cylindrospermopsin (CYN) in sediment cores, incorporating a variety of techniques, locations, and timescales. Different methods were used between studies for extraction and toxin analyses. Only selected studies have applied solid phase extraction (SPE) for concentration and purification (Table 1). It is generally advised to use solid phase based preparation techniques (such as SPE or solid phase adsorption toxin tracking technology) for improved cyanotoxin recovery [34].

**Figure 1:**
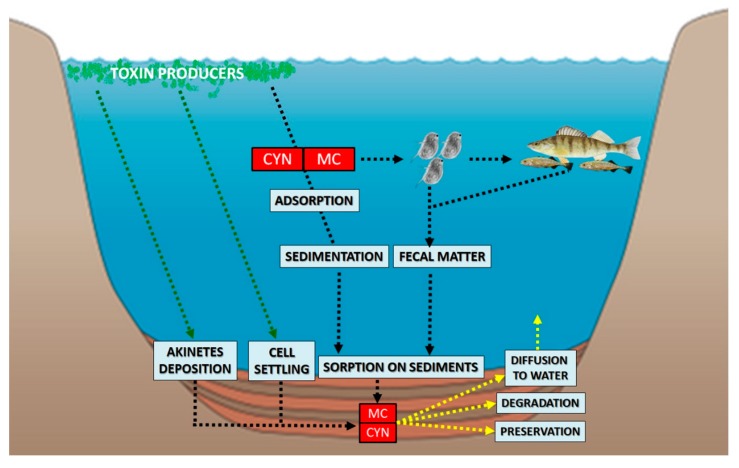
General pathways via which cyanotoxins can enter the lake sediments. MC: microcystin; CYN: cylindrospermopsin.

The toxin occurrence in the sediment record may occur via different pathways (Figure 1), among which the two main pathways are: (1) sorption of toxins and their delivery on to the lake bottom; and (2) the deposition of cells with internal toxins, or cells that are still capable of toxin production post deposition. As demonstrated, MC and CYN can undergo adsorption to natural organic matter, and thus, their sedimentation and further sedimentary burial is plausible [35,36,37,38]. On the other hand, selected cyanotoxin producers, such as *Microcystis* sp., can thrive at the lake bottom (e.g., overwinter in the temperate zone) [39], and evidence for the active production of MCs by these benthic populations has been presented, including cells buried in sediments up to six years [40]. Moreover, some CYN producers, belonging to *Oscillatoria* genus, are associated with benthos [41]; while selected planktonic species can also deposit their cells in sediments, with evidence of toxin production [42]. Under unfavorable conditions, selected toxin producers can form dormant cells known as akinetes, and deposit them in sediments. Whether these akinetes contain cyanotoxins has not been subject to investigation; but it was demonstrated that in some cases, up to several thousands of intact akinetes per g of sediment can be deposited [43]. If these cells contain selected toxins of interest, they would highly contribute to the sedimentary cyanotoxin pool.

Collectively, sedimentary cyanotoxin levels can reflect the release of these compounds into the water column, the deposition of their producers at the lake bottom, and further burial in the sediments. The studied toxin classes and the results of their quantification in sediments in the paleolimnological research are discussed in the following subsections.

### 2.1. Microcystins (MCs)

MCs are cyclic peptides containing two variable amino acids and aromatic 3-amino-9-methoxy-2,6,8-trimethyl-10-phenyldeca-4,6-dienoic acid (ADDA). To date, more than 250 congeners of MCs have been identified in cyanobacteria, with approximately 20% of them being likely the result of chemical or biochemical transformations. Alterations to MCs can occur in the environment or during sample handling and extraction of cyanobacteria, including oxidation products, methyl esters, or post-biosynthetic metabolites. The MC-LR (containing leucine and arginine) is the most often monitored and studied variant, and considered as the most toxic among MCs [44]. The main producer of these compounds is *Microcystis aeruginosa*, although a number of other freshwater cyanobacteria belonging to the *Anabaena/Dolichospermum*, *Nostoc*, *Oscillatoria*, and *Planktothrix* genera are capable of their biosynthesis [45]. MCs have been reported worldwide on every continent [46]. MCs are synthesized non-ribosomally via a thio-template mechanism involving multiple enzymes, e.g., peptide synthetases, polyketide synthases, putative transporter, and tailoring enzyme. The gene cluster of the MC biosynthesis pathway consists of ten genes (*mcyA-J*), with *mcyA* and *mcyE* used most often to identify the presence of toxin producers [47,48]. The ecological role of MCs is currently under debate, with multiple hypotheses proposed, including nutrient metabolism, iron acquisition, amelioration of oxidative stress, colony-formation, quorum-sensing, info-chemical signaling, participation in light adaptation, allelopathic interactions, and functional coupling with programmed cell death [14,49,50,51]. Due to their toxicity, MCs are currently the most studied and monitored cyanobacterial toxins. The main mechanism of their action involves the inhibition of protein phosphatases 1 and 2A in hepatocytes, which can eventually lead to liver damage. Moreover, MCs can potentially act as tumor promotors, as shown experimentally, and the International Agency on Cancer Research committee has concluded that MC-LR is possibly carcinogenic to humans (Group 2B) [52].

While paleolimnological techniques (photosynthetic pigments, akinetes) have been used to infer past cyanobacteria presence in lake systems [20,53,54,55,56], few studies have attempted to infer historic MC production. Cyanotoxin measurements in sediments are not common, but investigations have confirmed conditions that favor toxin preservation, as well as the potential use of fossilized toxins as a paleolimnological tool. In the case of MCs, their accumulation in sediments was first proven experimentally, and methods based on different analytical approaches to assess their sedimentary content were developed [57,58]. However, the partial degradation or transformation of MCs mediated by microbial activity must be taken into account, particularly under anoxic conditions which were demonstrated to stimulate their degradation [59]. It has also been proposed that MCs accumulating in sediments are not fully immobilized, and diffusion to the water column may occur [60]. Nevertheless, MC content has been measured in sediment cores from Polish lakes [61], Nebraska, USA [62], and Canada [60,63]; and, as demonstrated, the toxin production could be tracked over the past ≈150 years. Very few studies have attempted to reconstruct MC production prior to recent human development around lake systems (pre-150 years); most studies have utilized a variety of extraction techniques, and one of two analytical techniques: liquid chromatography–mass spectrometry (LC–MS), or gas chromatography–mass spectrometry (GC–MS) (Table 1 and Table 2). Recently, a longer history of potential MC production was constructed from akinetes, and by sequencing genes related to MC synthesis isolated from a sediment core from Lake Aydat, France [43]. While records of historic MC production are slowly being produced and timelines are being extended, a comprehensive extraction technique and a detailed understanding of MC degradation and transformation post-deposition have yet to be established.

As shown, the sedimentary MC content in the successive layers of sediment can demonstrate variability with no general trend of decrease [61,64]. This indicates that tracking their sedimentary content can be a reliable tool for following fluctuations in the development of MC-producers in the past. Coupling the MCs analyses with identification of algal pigments such as chlorophyll-a, zeaxanthin, and pheophytin-a in sediment cores can also be useful in reconstructing historical shifts in densities of MC-producing cyanobacteria, as shown in a study conducted on several lakes in Nebraska [62]. The observed levels of MCs in lake sediments are summarized in Table 2.

Only one study has addressed the relationships between sedimentary MC content and other parameters, potentially useful for understanding environmental conditions under which MC production is triggered. As demonstrated in Lake Baptiste (Canada), there was a strong positive correlation between total sedimentary MC levels, total Kjeldahl N, and total P inferred from diatom assemblages [63]. This clearly demonstrates that nutrients play a key role in the sedimentary accumulation of MC, and is consistent with observations in eutrophic lakes where usually higher water concentrations of toxins are observed [15,65]. However, increased nutrient levels are a result of favorable conditions for the proliferation of MC-producing cyanobacteria and do not add to elucidation of the biological function of MC. As previously discussed, various functions of this compound have been postulated. Evidence from some studies suggest that MC can be implicated in iron acquisition, since iron-depletion (but not nitrogen or phosphorus starvation) triggered its synthesis [16]. Therefore, it would be of interest to further investigate whether sedimentary Fe levels reveal any kind of relationship with accumulated MC levels. One should note, however, that Fe is a labile element and can undergo post-depositional mobility under anoxic conditions [66]. Therefore, its potential correlations with sedimentary cyanotoxin levels should be interpreted cautiously.

Most studies investigating sedimentary MC levels have only quantified total toxin content or specifically, the MC-LR variant (Table 1). Two studies, however, have quantified eight different MC congeners: MC-LR (and additionally -7dmLR), -RR, -YR, -WR, -LA, -LF, -LY, and –LW [60,63]. As shown, MC-LR and MC-LA were the dominant sedimentary forms, with the former revealing higher burial rates [60,63]. This is an interesting observation since MC-LA as well as other more hydrophobic MC variants appear to be less frequently found in aquatic environments, compared to MC-LR, MC-RR, and MC-YR [67,68]. However, MC-LA has been shown to be potentially more persistent in the environment [69,70]. The abovementioned observations highlight the need to investigate the various MC congeners in the total sedimentary toxic quota, particularly coupled with other abiotic and biotic parameters, to understand the potential environmental drivers affecting not only total MC production in aquatic environment, but also trends in the occurrence of its degradation and transformation post deposition.

### 2.2. Cylindrospermopsin (CYN)

CYN is a polyketide-derived alkaloid containing a central functional guanidine moiety combined with hydroxymethyluracil attached to its tricyclic carbon skeleton, and was first discovered in 1992 in the cyanobacterium *Raphidiopsis raciborskii* (formerly *Cylindrospermopsis raciborskii*) [71]. However, CYN was later confirmed to be produced by a number of other freshwater filamentous species belonging to the *Nostocales* and *Oscillatoriales* orders [3]. More recently, the terrestrial *Hormoscilla pringsheimi* was identified to be capable of CYN production [72,73]. To date, four other naturally occurring analogues have been identified in cyanobacteria: 7-epi-CYN, 7-deoxy-CYN, 7-deoxy-sulfate-CYN, and -deoxy-desulfo-12-CYN [74,75], with CYN being the most toxic. The biological role of CYN is under debate, with experimental studies suggesting its involvement in the acquisition of inorganic phosphate via inducement of alkaline phosphatase in other photosynthetic microalgae [12,76,77]. The biosynthesis of CYN is related to the *cyr* gene cluster, spanning 43 kb and comprised of 15 open reading frames, containing the genes required for biosynthesis, regulation, and transport of the compound [78]. Assessment of the ability of strains to produce CYN is usually performed by amplification of the *cyrJ* encoding sulfotransferase, essential for tailoring a final reaction of biosynthesis [79,80]. As extensively shown, CYN production is highly strain-dependent but geographically extends throughout the globe, encompassing Australia, New Zealand, Antarctica, Asia, South and North America, and Europe [3,81]. A number of its potential producers, such as *R*. *raciborskii* and *Chrysosporum ovalisporum*, are capable of forming blooms at different locations and under favorable conditions, such as eutrophication and increased water temperature [82,83]. As shown, CYN reveals a wide range of cytotoxic activities in vitro and in vivo [84] and was suggested as a causative factor of human poisoning on Palm Island, Australia in 1978 [17]; and, along with MCs, in fatal cases of patients undergoing treatment in a dialysis center in Caruaru, Brazil in 1996 [85].

CYN is a relatively stable compound under various environmental conditions, including visible and UV light, and over a wide range of pH levels and temperatures [31,86,87]. Its degradation can also be inhibited under anoxia [88]. Moreover, CYN has been shown to be highly mobile during sediment passage, while its sorption is dependent on organic carbon availability [89]. Altogether, this highlights that CYN could be a potential target for paleolimnological studies.

So far, only one study has approached the identification of CYN in core sediments. The investigation performed by Waters [33] aimed to measure its content in Lake Griffin (Florida, USA) sediments up to 300 cm in depth, representative of the last 4700 years, as dated using the ^210^Pb record and calibrated AMS ^14^C [33]. Following the extraction throughout the core using a 1:1 mixture of methanol/HPLC-grade water, the study employed a commercial immunoassay microtiter plate kit, based on the recognition of CYN by a specific antibody that eventually generates a product which can be quantified spectrophotometrically. This assay cannot distinguish between CYN analogues, and gives an estimate of the total CYN level in the studied samples. Moreover, according to information given by the manufacturer, test interferences caused by matrix effects cannot be completely excluded. The results of CYN levels in sedimentary samples yielded from this assay are yet to be verified by alternative analytical techniques of higher selectivity, such as high-performance liquid chromatograph (HPLC) or LC-MS.

In the shallow, sub-tropical Lake Griffin, Waters [33] divided the investigated sediment core into three zones according to CYN abundance, which represented: (1) mid-Holocene period (mean content 1.4 ng/g org); (2) mid to late Holocene prior to human settlements (0.87 ng/g org); and (3) the 150–200 years before present, when human impacts on the lake were already present (2.61 ng/g org). Increased CYN levels in the third zone indicate that human-driven eutrophication has resulted in the increase of the occurrence of CYN-producers in the studied lake. These cyanobacteria were apparently present long before any ecosystem disturbance was exerted by human activities [33].

In addition to CYN quantification, the study by Waters [33] also addressed the contents of organic matter, nutrients, and photosynthetic pigments in core sediments, allowing testing to determine whether any significant correlations between these parameters and toxin levels were detectable. As shown using PCA analysis, CYN levels in the sediments representing the period of human impact on Lake Griffin correlated positively with various photosynthetic pigments. This highlights that the increased toxin content in these sediments is an indication of lake eutrophication; while its lower level, in sediments predating human impacts, is not a result of slow compound degradation occurring over time. As suggested, the lowest CYN levels found in sediments representing the mid-Holocene period correlated with low N/P values, which may be an indication that limited N availability in the lake triggered CYN production [33]. However, experimental data suggests that under N limitation, the release of the toxin is highly suppressed, while additional nitrate supply highly promotes this process [90]. On the other hand, N depletion was demonstrated to cause an increase in intracellular pool [90]—it cannot be ruled out that N-starved cyanobacteria cells deposited in sediments contributed to sedimentary pool of CYN. In contrast, there is experimental evidence that P starvation may induce CYN synthesis and release [76,90,91]. No apparent association of that kind between sedimentary P and CYN levels were found in Lake Griffin. However, low-CYN sediments contained the lowest P content [33]. It should be stressed that sediment P may not necessarily reflect past water column concentrations due to post-depositional transformations and mobility, and the reduction of Fe and subsequent dissolution of the Fe-P complexes [66]. These issues require further elucidation in other paleolimnological surveys.

## 3. Establishment of Cyanotoxins as a Paleolimnological Tool

Although cyanotoxins show promise as a potential paleolimnological tool, research has not been conducted to calibrate and validate sediment core measurements to water column toxin production, to identify the primary pathways and drivers of the degradation of sedimentary cyanotoxins (Table 3). Given that MC and CYN possess multiple congeners, post depositional transitions need to be established, so that degradation pathways can be applied to sediment core interpretation [92]. In addition, most sedimentary cyanotoxin investigations have used a variety of extraction protocols, including mixtures of methanol, water, pH, acids, and other combinations to extract sedimentary toxins [33,60,61,62,63]. Recently, the cyanotoxin analytical company, Abraxis, Inc. (Warminster, PA, USA; www.abraxiskits.com), produced a soil protocol for ELISA techniques, but whether this technique works in ancient sediments is yet to be determined or validated. As a result, future investigation on using MC and CYN as paleolimnological tools should include information beneficial to toxin stratigraphic interpretation, in addition to relative change through time.

It is highly advised, to employ advanced analytical tools in the identification of cyanotoxins in core sediments, since immunoassays may reveal some cross-reactivity particularly in an enriched organic matrix. For example, as shown in the case of CYN, false positive results could not be discounted when using commercial ELISA kit [93]. Methods based on HLPC, LC/MS, or GC/MS have been developed, including approaches that allow for simultaneous detection of multi-class cyanobacterial compounds in one run [94]. This will enable the history and dynamics of the production of various cyanotoxins to be tracked using sediment cores. However, the simultaneous detection of more than one cyanotoxin was previously applied to relatively enriched matrices (e.g., vegetables) [95], thus their application to core sediments would first require analytical method validation to ensure accuracy, specificity, and reproducibility. Currently there are no reference materials for cyanotoxins in lake sediments, thus the validation of such a method would be based on spike additions using compound standards.

## 4. Future Research Prospects

Apart from MCs and CYN, a number of other cyanobacterial metabolites have been shown to exhibit different degrees and mechanisms of toxicity. Neurotoxic alkaloids, anatoxins (ATXs), saxitoxins (STXs), neurotoxic non-proteinogenic amino acid beta-methylamino-L-alanine (BMAA), and hepatotic cyclic peptides nodularins (NODs) have received the most attention and are of the greatest concern to animals and humans [6,7]. The latter group of compounds, NODs, comprising of 10 variants discovered to date, are highly related structurally to MCs [96] and reveal a similar mechanism of toxic action mediated through the potent inhibition of protein phosphatases in hepatocytes [6]. So far, *Nodularia spumigena*, associated with brackish (e.g., Baltic Sea) and saline waters, and benthic saline *Nostoc* sp. are the only identified producers of NODs [97]. To date, 10 structural variants have been discovered. Considering that MCs were successfully identified in core sediments, it is plausible that NODs could also be quantified. Indeed, it was initially shown by a survey conducted in the Baltic Sea that NODs are detectable in the uppermost layer of the sediments using LC-MS, with two demethylated forms successfully identified: [DMAdda3]NOD and [dhb5]NOD [98]. This further supports the rationale for investigations of NOD levels in core sediments to track the history of its production in ecosystems such as the Baltic Sea.

Metabolites such as ATX have been determined to be unstable in the water column and degrade rapidly, independent of oxygen availability, under UV radiation and microbial activity [99,100]; thus its occurrence in sediments is unlikely. Conversely, the by-products of ATX conversion, such as dihydroATX and epoxyATX, are shown to be more stable (while much less toxic) than the parent compound [101]. The presence of these variants in sediments and the analysis of their concentrations, requires further study. It has also been postulated that BMAA, due to its chemical structure, is readily biodegradable and would not accumulate in sediments at detectable levels [102]. Investigations by Jiang et al. (2008) did not find sedimentary BMAA, even though it was present in planktonic samples [103]. Notably, it was shown that BMAA is not exclusively produced by cyanobacteria, freshwater diatoms were also shown to be capable of its production [104]. Therefore, even if it is quantified in sediments, elucidation of its potential source of origin would require simultaneous determination of cyanobacterial and diatomaceous subfossils.

There is no data on STXs accumulation in sediments, although it was documented experimentally undergoing adsorption to sediments [105]. These compounds are known to be exclusively synthesized by cyanobacteria (belonging to the *Anabaena*, *Raphidiopsis*, *Aphanizomenon Planktothrix*, and *Lyngbya* genera) in freshwater ecosystems (although in marine environments, selected species of dinoflagellates are their major producers) [106]. However, it was also shown that different variants of STXs (saxitoxin, neosaxitoxin, gonyautoxins 2–3, and icarbamoyl-gonyautoxins 2–3) are fully biodegradable within a few days by some bacterial species isolated from digestive tracts of blue mussels [107]. Whether similar processes occur in sediments remains yet to be investigated. While it remains to be determined whether these cyanotoxins can be quantified directly in core sediments, some cues on their production could be drawn from studies of ancient DNA, and by targeting the genes responsible for their biosynthesis.

There are a number of other cyanobacterial metabolites that occur in aquatic ecosystems in need of investigation concerning pathways of synthesis and toxicity. Overall, greater than 50 bioactive classes produced by cyanobacteria have been identified on the basis of their chemical properties (as reviewed by [108]. Besides the compounds already discussed in this review, the following molecules were attributed to freshwater cyanobacteria: aeruginosin, anabaenopeptin, cyanopeptolin, calothrixin, carmabin, microcyclamide, microginin, microguanidine, microviridin, and polymethoxy-1-alkenes. The latter was shown to reveal teratogenicity in vivo, and produced by selected cyanobacteria, as well as green algae [109,110]. Whether PMAs and other abovementioned metabolites are stable enough to accumulate in sediments is yet to be elucidated. However, it is plausible that they will not be persistent since alkenes are generally prone to rancidification due to a lack of full saturation of the lipid molecules, and unsaturated fatty acids are known to be susceptible to rancidity [111].

It is also unknown whether cyanobacterial akinetes can retain cyanotoxins, and whether this is the case for akinetes deposited in sediment layers. So far, this has not been a subject of any study. However, paleolimnological investigations have already demonstrated that akinetes can be found in sediments, their characteristic morphological features can be used to identify the species they originated from, and toxin biosynthesis genes can be successfully amplified from their DNA [43,56,112]. If akinetes of toxin-producing strains contain cyanotoxins, then they may make a significant contribution to the sedimentary pool of these compounds, given the fact that in some cases, a high number of these cells can be identified in sediment layers. For example, Legrand et al. [43] reported up to 705,000 and 36,400 akinetes of *Dolichospermum macrosporum* and *D. flos-aquae* per g of dried sediment, respectively.

While initial successes have shown that cyanotoxins are a potential paleolimnological tool, multiple developments in experimental methods and interpretations are still needed (Table 3). This list is not exhaustive, and demonstrates a focused effort to develop this very important application of cyanotoxin research. In addition, we highlight unique data that demonstrates potential novel questions that sedimentary cyanotoxin measurements could pose (Table 3). By coupling these paleolimnological investigations with modern research, a more comprehensive understanding of cyanotoxin ecology and triggers, could potentially be obtained.

Furthermore, cyanotoxins and other factors from paleolimnological studies would support other investigations focusing on the elucidation of their ecological roles, which, as already outlined in this review, is still not well understood. Simultaneous analyses of chemical composition (e.g., content of N, P, and Fe), subfossils of phyto- and zooplankton, and ancient DNA, would determine the relationship between these parameters and cyanotoxin levels. A number of investigations have suggested that these compounds can be implicated in nutrient acquisition, defense against grazers, and allelopathic interactions with phytoplankton [1,12,51]. Even though such investigation would not imply any causation, they could be valuable for the interpretation of experimental studies on the biological role of cyanobacterial metabolites.

## 5. Conclusions

At the present time, paleolimnological studies on cyanotoxin levels in sediments are still scarce, and limited only to two classes of compounds: MCs and CYN. While cyanotoxins can be extracted and measured from sediments, little is known on post-depositional transformations, or on linkages between sedimentary toxin amounts and water column production. It is clear that such an approach can provide interesting insight on the dynamics of cyanobacterial metabolites of the past, including periods predating human impacts on aquatic environments. Furthermore, such investigations can be coupled with analyses of ancient DNA isolated from sediments as well as other abiotic and biotic sedimentary features, to produce valuable data on cyanotoxin occurrence and additional clues on their ecological roles. While multiple questions still remain concerning paleolimnology and cyanotoxins, the potential information that could be ascertained from sediment reconstructions would provide valuable data and insight into questions that have remained elusive in experimental and monitoring investigations.

## Figures and Tables

**Table 1:** Summary on cyanotoxin paleolimnological studies.

Toxin	Location	Core Length ^1^	Oldest Date ^2^	Detection Method	Extraction	Reference
MC (8 congeners)	Canada, Lake Baptiste	50	1824 AD	LC-MS	75% MeOH, SPE	[63]
MC-Total	Poland, Lake Glębokie	40	n.a.	GC-MS	75% MeOH	[61]
MC-Total	Poland, Lake Syczyskie	50	n.a.	GC-MS	75% MeOH	[61]
MC (8 congeners)	Canada/USA, Lake of the Woods	7	2000 AD	LC-MS	75% MeOH, SPE	[60]
MC-Total	Poland, Lake Syczyńskie	50	1800s?	GC-MS	75% MeOH	[64]
MC-LR	USA (Nebraska), Two Mile Lake	18	1866 AD	LC-MS	EDTA, Na_4_P_2_O_7_, MeOH, SPE	[62]
MC-LR	USA (Nebraska), Lake Dewey	40	1945 AD	LC-MS	EDTA, Na_4_P_2_O_7_, MeOH, SPE	[62]
MC-LR	USA (Nebraska), Island Lake	20	1832 AD	LC-MS	EDTA, Na_4_P_2_O_7_, MeOH, SPE	[62]
CYN	USA (Florida), Lake Griffin	300	4732 BP	ELISA	50% MeOH	[33]

n.a.: not available; SPE: solid phase extraction; ^1^ core length is in cm and represents the deepest depth where cyanotoxins were detected; ^2^ oldest date represents the oldest datable section of the core where cyanotoxins were detected.

**Table 2:** Levels of cyanotoxins detected in paleolimnological studies.

Cyanotoxins Reported	Units	Max Value Post-1980 AD	Max Value Pre-1980 AD	Method of Detection	Reference
CYN	ng g^−1^ org. matter	7	4	ELISA	[33]
MC-LR	µg cm^−2^ yr^−1^	0.045	0.015	LC-MS	[62]
MC (8 congeners)	ng g^−1^ dry weight	>1000	70	LC-MS	[63]
MC - total	µg g^−1^ dry weight	900	100	GC-MS	[61]

MC: microcystin; CYN: cylindrospermopsin.

**Table 3:** The summary of top developments needed to establish cyanotoxins as a paleolimnological tool and a list of unique applications that paleolimnological studies on cyanotoxins can provide for environmental sciences.

**Required Developments**
Improved extraction techniques for toxins in sediments considering measuring technique and sediment type. Linkages between sediment cyanotoxin concentrations and water column cyanotoxin production. Post-depositional cyanotoxin transformations and degradation pathways in sediments. Development of paleolimnological applications of other cyanotoxins, in addition to MCs and CYN. Comparison of sedimentary cyanotoxins with other paleolimnological measurements.
**Unique Applications**
Identification of environmental triggers for cyanotoxin production. Cyanotoxin dynamics in response to regime shifts, cultural eutrophication, fishery alterations, and land use change. Cyanotoxin production prior to human disturbances. Cyanotoxin storage in sediments with potential of reintroduction during mixing events and sediment resuspension.

MC: microcystin; CYN: cylindrospermopsin.
